# Supplementary material for: Morphine suppresses peripheral responses and transforms brain myeloid gene expression to favor neuropathogenesis in SIV infection
Source: Front Immunol. 2022 Nov 16;13:1012884. doi: 10.3389/fimmu.2022.1012884 (PMC9709286; doi:10.3389/fimmu.2022.1012884)
Supplement: Supplementary file 1 [file DataSheet_1.docx]

**Supplemental Table Legends**

**Supplemental Table 1. Plasma biomarkers.** Values before and after morphine administration for the morphine group, and before and 2, 5, 22, and 35-weeks after SIV inoculation are given for all monkeys. For statistical purposes values <LLOQ were one-half of the LLOQ.

**Supplemental Table 2. DEGs between the clusters.** FDR q-value, fold-change, and least-square mean (LSMean) values for each cluster compared to the other 5 clusters. Each cluster is shown on a separate worksheet, DEGs lists were filtered for an FDR <0.01, fold change >|1.5|and a minimal LSMean expression of at least 50 in either the indicated cluster or the combination of the other clusters.

**Supplemental Table 3. DEGs within each cluster comparing the morphine group to the saline group.** The rhesus gene IDs and the human homologues are indicated, along with the FDR q-value, fold-change, and least-square mean (LSMean) values for the morphine group and the saline group within each cluster. Each cluster is shown on its indicated worksheet, DEGs lists were filtered for an FDR <0.01, fold change >|1.5|and a minimal LSMean expression of > 50 in either the morphine or saline group within the cluster.

**Supplemental Table 4. Gene sets used for GSEA.** The published lists of human genes for the gene sets are listed, followed by the mapping to rhesus homologues. The gene names highlighted in grey code for ribosomal proteins that were not considered in the GSEA.

**Supplemental Table 5. IPA analysis.** The canonical pathways assessed for the DEGs found due to morphine treatment are shown on a separate worksheet for each cluster. Highlighted are those with a p-value <0.05 and a Z-score> 1.
