# Supplementary material for: Morphine suppresses peripheral responses and transforms brain myeloid gene expression to favor neuropathogenesis in SIV infection
Source: Front Immunol. 2022 Nov 16;13:1012884. doi: 10.3389/fimmu.2022.1012884 (PMC9709286; doi:10.3389/fimmu.2022.1012884)
Supplement: Supplementary file 2 [file DataSheet_2.docx]

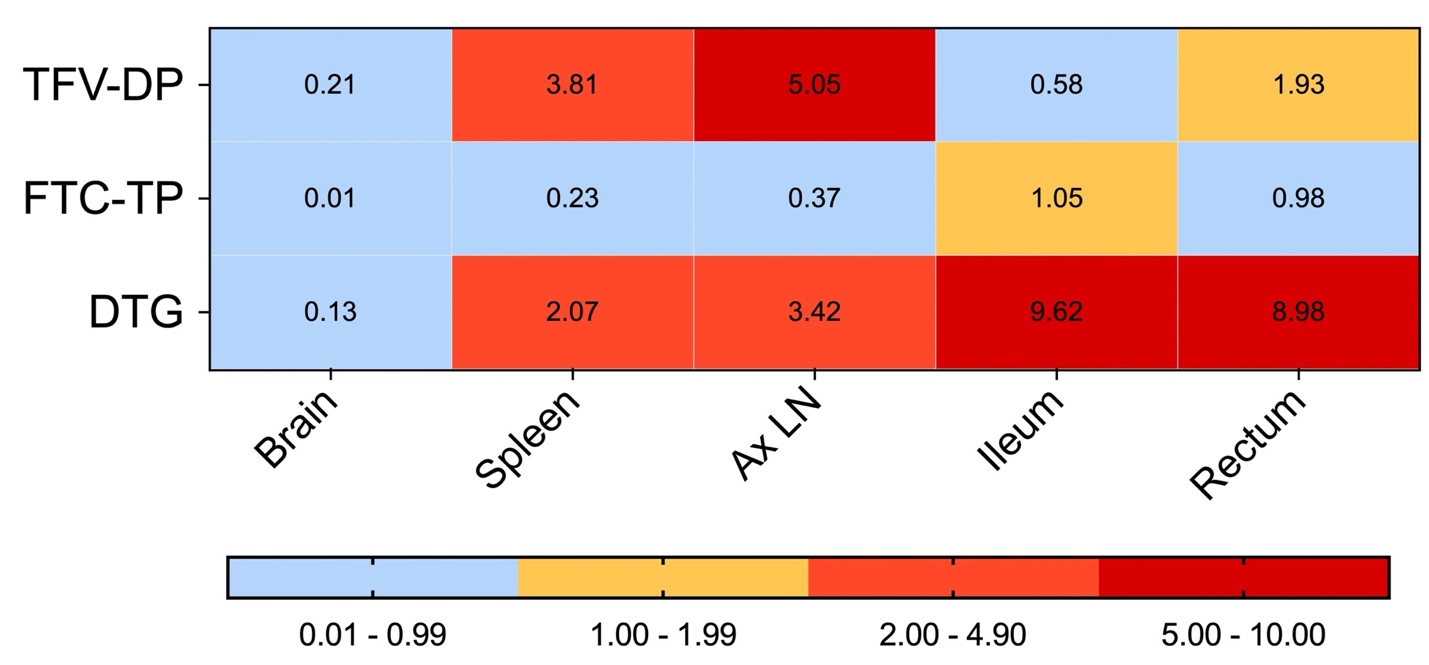


**Supplemental Figure 1. Ratio of tissue concentration of active ARV moiety to the IC_50_ (TFV-DP and FTC-TP) and IC_90_ (DTG).** The concentrations in the 5 regions of the brain were averaged for the brain value.

**
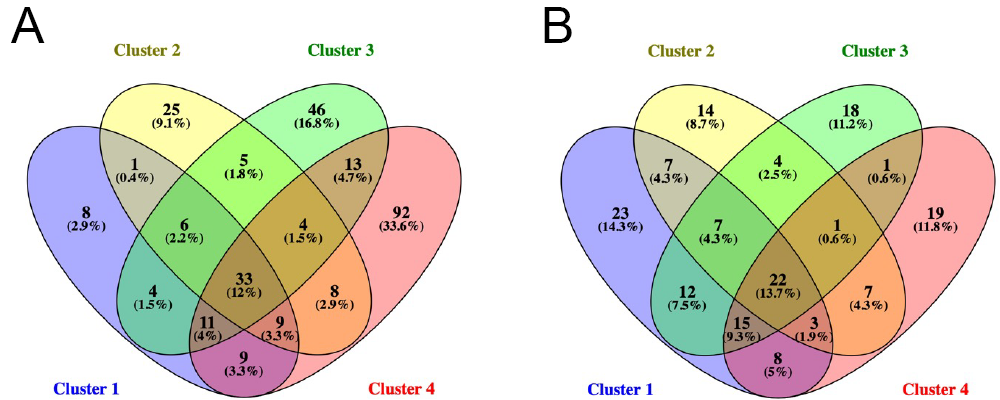
**

**Supplemental Figure 2.** **Common genes are differentially regulated by morphine in microglia clusters.** Venn diagram showing the intersections of the **A)** up-regulated and **B)** down-regulated (right) DEGs found in microglia clusters 1-4.
